# Effects of additives and electrolytic treatment to remove tritium from contaminated water

**DOI:** 10.1016/j.heliyon.2023.e17031

**Published:** 2023-06-11

**Authors:** Shizutoshi Ando, Takashi Komatsuzaki, Mitsukiyo Okada, Noriaki Kataoka

**Affiliations:** a Department of Electrical Engineering, Tokyo University of Science, 6-3-1 Niijuku, Katsushika-ku, Tokyo, 125-8585, Japan; b Gabriel Co., Ltd., 4-5-10 Nishi-shinjuku, Shinjuku-ku, Tokyo, 160-0023, Japan; c Research and Development Department, Tokyo Metropolitan Industrial Technology Research Institute, 2-4-10 Aomi, Koutou-ku, Tokyo, 135-0064, Japan

**Keywords:** Radioactive contamination water, Tritiated water (HTO), Electrolytic treatment, Carbide powder, Silica stone, Tritium recovery, Efficiency of tritium separation (HTO/H_2_O)

## Abstract

As a method for separating tritiated water from radioactively contaminated water, an additive was mixed into the contaminated water and their treatments of agitation/circulation and electrolytic was considered to improve of the separation efficiency. Carbide powder and ore (silica stone) powder were used as additives. The radioactivity concentration of the tritium-contaminated water (tritiated water; HTO) was 366 Bq/mL before treatment, however it obtained 333 Bq/mL and decreasing rate of 9.02%, and a high separation efficiency after treatment. Furthermore, in the reproducibility experiments (five times) using high content of HTO, the average HTO/H_2_O separation efficiency was obtained about 5.59% indicating good reproducibility. The agitation/circulation treatment process was dissociated and ionized HTO, and was prepared colloidal particles by OT^−^ and ^3^T^+^. In the electrolytic treatment process, the colloidal particles in HTO were deposited on the both electrodes applied DC voltage, and was efficiently removed tritium. These treatment processes using additives were found to be useful for removal of tritium.

## Highlights

• Agitation/circulation processing and electrolytic treatment were suggested to improve HTO/H__2__O separation efficiency.
• It was obtained a high HTO/H_2_O separation efficiency of 9.02%.
• Carbide and ore (silica stone) powders as additive were useful for adsorbents of tritium water.

## 1 Introduction

The 2011 off the Pacific coast of Tohoku Earthquake (the Great East Japan Earthquake) and subsequent tsunami occurred on March 11, 2011 caused enormous damage. In particular, the meltdown at the Fukushima Daiichi Nuclear Power Station developed into a nuclear accident involving the leakage of a large amount of radioactive material and was discharged a large amount of highly radiation-contaminated water. This radioactively contaminated water contains strontium 90 (^90^Sr), iodine 131 (^131^I), cesium 134 and 137 (^134^Ce and ^137^Ce) and tritium (^3^H) and is treated in the multi-nuclide removal system (ALPS; Advanced Liquid Processing System) [1]. However, tritium is much more difficult to remove than other radioactive materials, and more than 1.2 million tons of treated water (highly tritium-contaminated water) after purification is stored in storage tanks (approximately 1000 tanks), and the amount continues to increase.

Tritium under the contamination disposal exists as tritiated water (HTO), and there are several tritium separation (HTO/H_2_O separation) techniques from water, including distillation method [2], electrolysis method [3] and isotope exchange method [4]. Ichimura et al. reported on the photolysis of tritiated water by Pt/TiO_2_ photocatalysis [5]. Ihara et al. reported the efficient separation of tritiated water from contaminated water using the sintered aluminum powder porous filter [6]. In addition, a method using spinel structure oxide compounds as a tritium adsorbent has been reported as a safe and easy-to-operate tritium removal technology for the large amount of radioactive contaminated water (tritiated water) discharged from the Fukushima Daiichi Nuclear Power Station [7].

In this study, the effects of adding carbide powder and ore (powder) to tritium-contaminated water (HTO-containing water) as tritium adsorbents was investigated. Furthermore, we aimed to improve the efficiency of HTO/H_2_O separation by stirring/circulating treatment and electrolytic treatment the added tritium-contaminated water.

## 2 Experiment

**Fig. 1:**
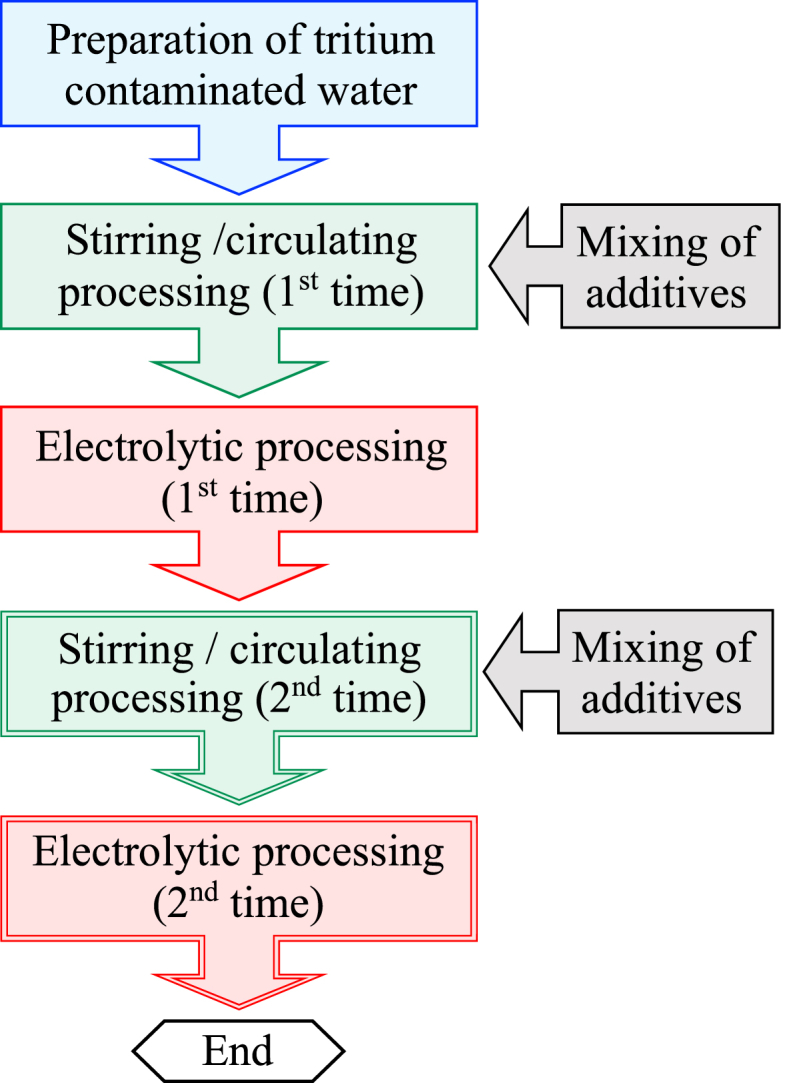
The processing procedure of tritium-contaminated water.

The process flow diagram for treatment of tritium-contaminated water is shown in Fig. 1. Tritium pseudo-contaminated water was prepared by diluting HTO-containing water with purified water. Tritium pseudo-contaminated water was treated by adding carbide powder solution and ore powder, stirred/circulated, and subsequently electrolysis. Details of the preparation of contaminated water and additives, stirring/circulating treatment, and electrolysis treatment are described in the next section.

### 2.1 Preparation of tritium-contaminated water and additives

HTO-containing water of 37 MBq/mL and 32 kBq/mL produced by ATR (American Radiolabeled Chemicals, Inc.), respectively, with 0.35 mL and 0.65 mL were diluted with purified water of 50 L in order to prepare as tritium pseudo-contaminated water with 481.64 Bq/mL (theoretical calculation value). Highly concentrated tritium pseudo-contaminated water of 1013 Bq/mL (theoretical calculation value) was also prepared by diluting 1.35 mL of HTO-containing water (37 MBq/mL) produced by ATR to purified water of 50 L.

**Fig. 2:**
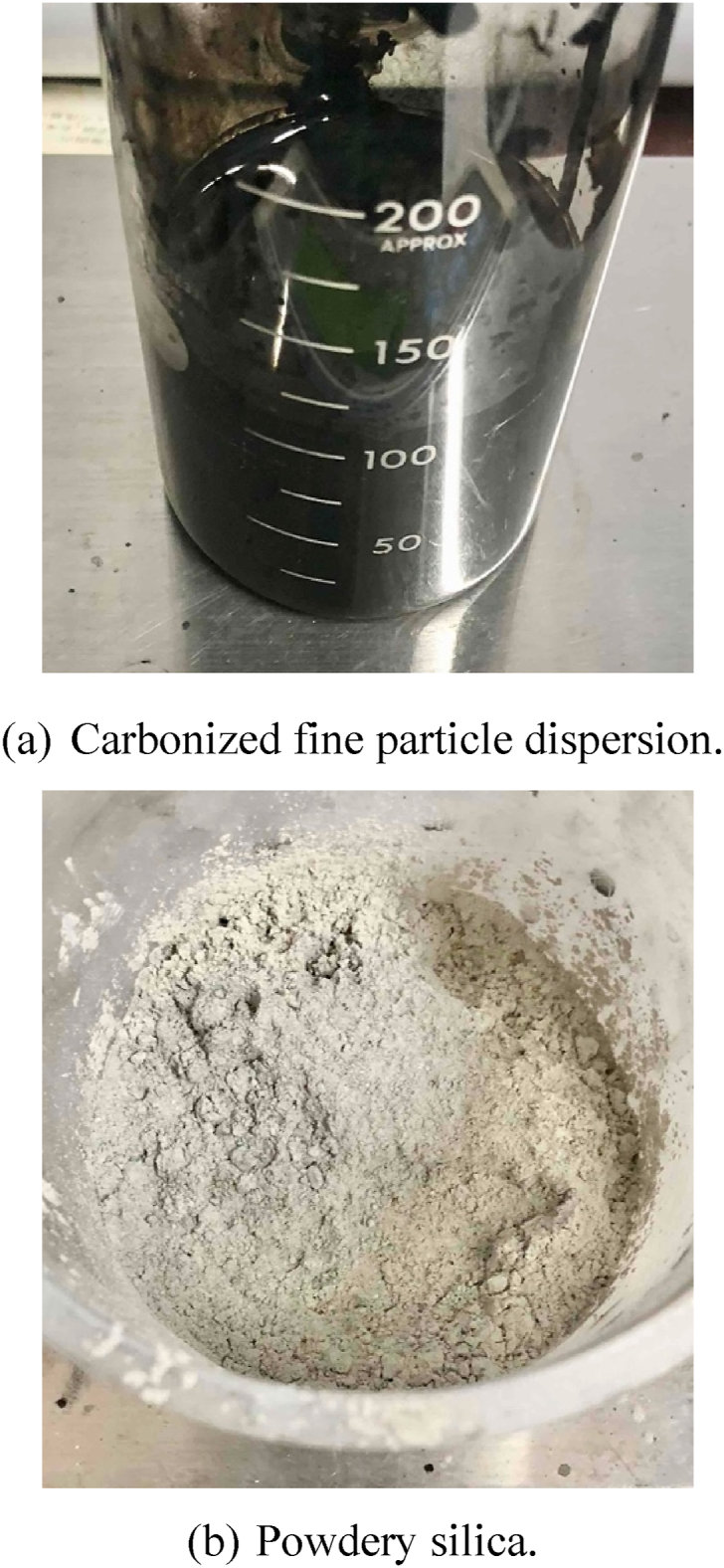
Carbonized fine particle dispersion and powdery silica used as additives. (a) Carbonized fine particle dispersion. (b) Powdery silica.

Additives were used Carbide powder dispersion and silica ore powder as shown in Fig. 2. The carbide powders were prepared by burning rice husks, flowers, wood, and grass at 400–1200 °C under oxygen-free atmosphere (as activated carbon), and then pulverized to less than 100 nm in size. Carbide powder of 10 g was added to purified water of 100 mL, and the added solution of 30 mL was further diluted to purified water of 70 mL to prepare the carbide powder dispersion of 100 mL. The mineral was silica ore, which was pulverized and powdered in the same manner as the carbide powder. These additives were intended to adsorb OT^−^ and ^3^T^+^ that HTO were dissociated and ionized by various treatment processes.

### 2.2 Treatment methods for tritium-contaminated water

#### 2.2.1 Stirring/circulating treatment process

**Fig. 3:**
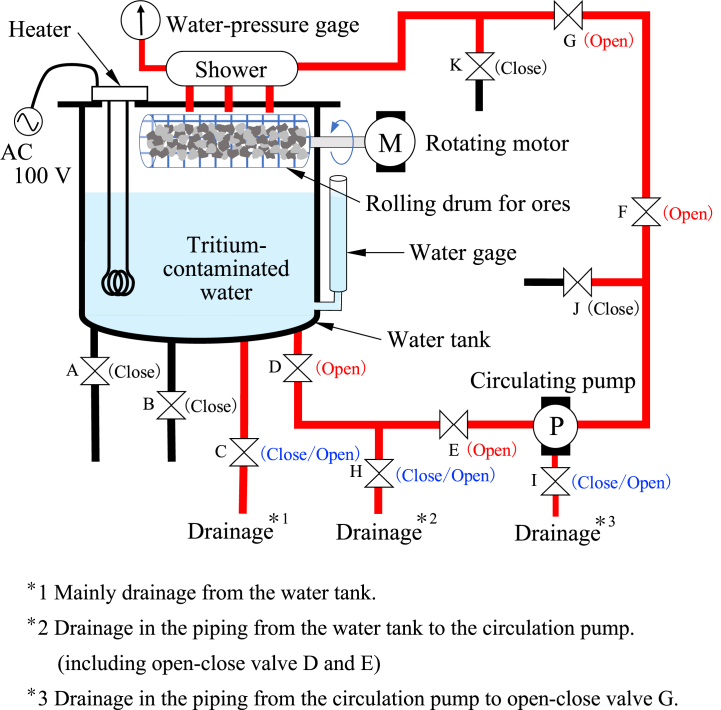
The apparatus of stirring and circulating treatment for tritium-contaminated water.

The stirring/circulating treatment is performed by ATRAS III (Gabriel Co., Ltd.), and a schematic of the apparatus is shown in Fig. 3. 50 L of tritium pseudo-contaminated water, 100 mL of carbide powder dispersed water, and 100 g of ore powder were fed into a water tank, and the contaminated water in the tank was heated to 50 °C by a heater, then stirred and circulated by a circulation pump. After 30 min, another 100 mL of carbide powder dispersed water and 100 g of ore powder were added to the tank and continuously stirred and circulated for 30 min (total 1 h). The rotating drum was filled with 1–2 cm uncrushed ore (silica) and was rotated during treatment, and contaminated water circulated from the water tank was sprayed into the rotating drum from a shower and returned to the water tank.

For the 2nd treatment, as shown in Fig. 1, after the first electrolysis treatment, 30 L of contaminated water, 100 mL of carbide powder dispersion water, and 100 g of ore powder were again fed into the water tank of ATRAS III, and the same procedure as for the 1st stirring/circulating treatment was used thereafter.

#### 2.2.2 Electrolytic treatment process

**Fig. 4:**
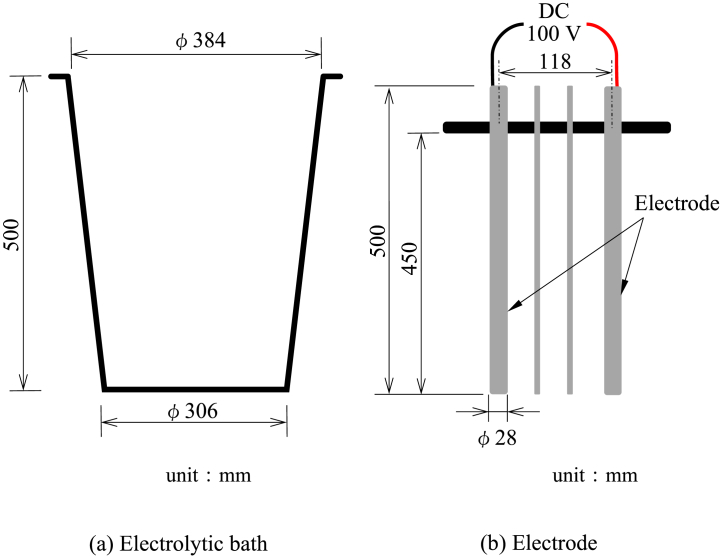
The apparatus of electrolytic treatment for tritium-contaminated water. (a) Electrolytic bath (b) Electrode.

The electrolytic treatment was performed by ATRAS Mini (Gabriel Co., Ltd.), a schematic of the apparatus is shown in Fig. 4. The electrode materials were made of stainless steel round bar (SS400, φ28 × 500 mm) as the cathode and aluminum round bar (φ28 × 500 mm) as the anode. After the 1st stirring/circulating treatment, 30 L of treated water was fed into the electrolytic bath of the ATRAS Mini and electrodes were installed. DC voltage of 100 V was applied between the electrodes, and electrolytic treatment was performed for 24 h.

For the 2nd electrode treatment, 30 L of treated contaminated water was again fed into the electrolytic bath after the 2nd stirring/circulating treatment, and DC 100 V was applied between the electrodes and the electrolytic treatment was performed for 5 h.

### 2.3 Measurement of tritium concentration

The radioactivity concentration of tritium in contaminated water was referred to the atmospheric distillation method of the MEXT Radioactivity Measurement Method Series 9 ″Tritium Analysis Method” as a pretreatment. 20-mL low potash glass vials was used as the measurement vessel and Ultima Gold LLT (10 mL) as the scintillator. After mixing, tritium concentration was measured with a low background liquid scintillation counter.

## 3 Results and discussion

**Fig. 5:**
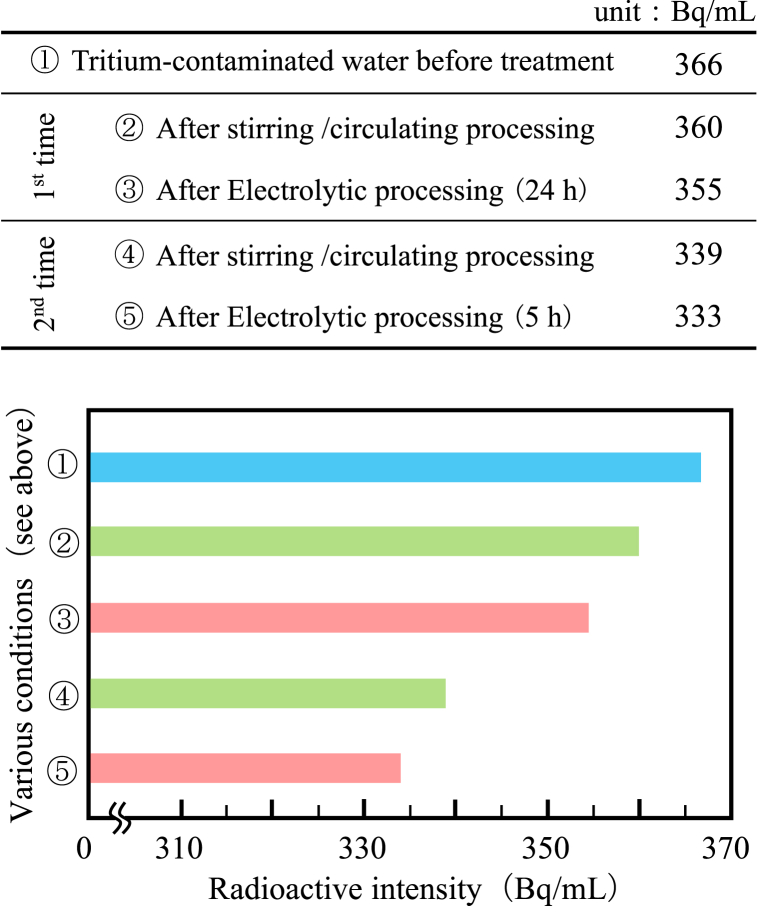
The radioactive concentration of tritium-contaminated water after the various treatments.

The table that changes in the radioactivity concentration of tritium-contaminated water after the addition of carbide powder dispersion solution and ore powder to prepared tritiated pseudo-contaminated water, after stirring/circulating treatment, and electrolysis treatment and their graphs are shown in Fig. 5. First, the radioactivity concentration prepared tritium pseudo-contaminated water (50 L) was 366 Bq/mL. This measured value was used as the initial value (reference value) before treatment. The radioactivity concentration of the tritium-contaminated water after the 1st stirring/circulating treatment was 360 Bq/mL, indicating an attenuation of about 1.64%. In this treatment, carbide powder dispersion and ore powder were added to contaminated water twice, with the aim of adsorbing tritiated water to these additives. The tritium radioactivity concentration of the silica stone in the rotating drum after treatment was measured to be 3.71 Bq/g. Therefore, this decreasing rate can be attributed partly to the adsorption of the silica in the ore drum. Adsorption of tritium on carbide powder and silica ore (lump and powder) mixed as additives also can be considered. These adsorptions are considered to be the adsorption of ^3^T^+^ by the following reactions of tritium ions (^3^T^+^) and hydroxide ions containing tritium ions (OT^−^), similar to the results described in the published patent bulletin by Koyanaka [8].

Eq. 1 OT^−^ → ^3^T^+^ + (1/2) O_2_ + 2e^−^

Generally, tritium in water exists as water molecules (T_2_O and HTO), and it is reported that the reaction in Eq. (1) causes the following dissociation reaction of tritium molecules, and all chemical species of tritium existing in water are adsorbed and separated from the water to the adsorbent [9].

Eq. 2 T_2_O → ^3^T^+^ + OT^−^

HTO → H^+^ + OT^−^ Eq. 3

Eq. 4 HTO → ^3^T^+^ + OH^−^

The radioactivity concentration of the tritium-contaminated water after the 1st electrolysis treatment was 355 Bq/mL and further decreased by about 1.38% (about 3.1% above the reference value).

**Fig. 6:**
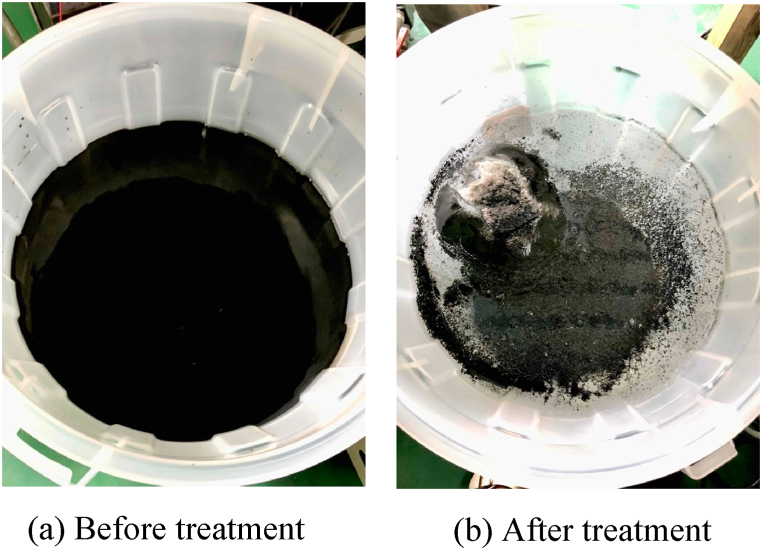
Tritium-contaminated water in the electrolytic bath with (a) before and (b) after electrolytic processing (1st time). (a) Before treatment (b) After treatment.

**Fig. 7:**
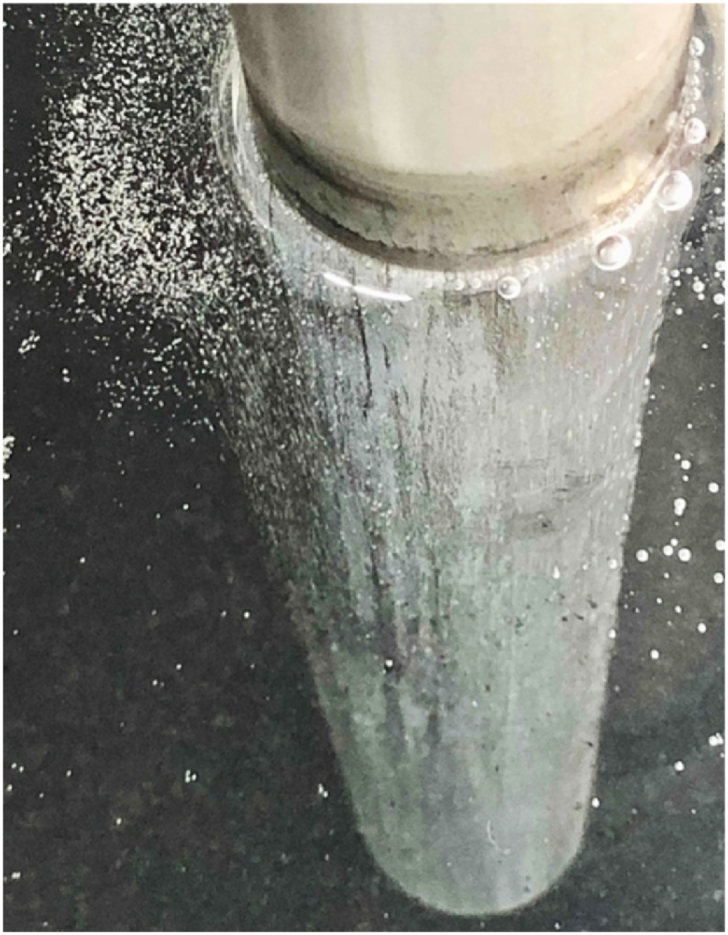
The state of generated gases around electrode during the electrolytic processing of tritium-contaminated water.

Fig. 6 shows the tritiated water in the electrolytic bath before and after the electrolysis treatment. The tritiated water before the electrolysis treatment was black in color because the carbide powder dispersion solution was sufficiently stirred in the previous process. On the other hand, in the tritiated water after electrolysis treatment, the added carbide powder dispersion solution was separated and agglomerated, resulting in a clear and colorless solution, while the carbide powders and silica powders precipitated at the bottom of the electrolytic bath (some of them floated on the water surface). As shown in Fig. 7, many bubbles were observed in the tritiated water near the (both) electrodes during the electrolysis treatment.

In the electrolysis of water containing tritium, it is known hydrogen ions (oxonium ions: H_3_O^+^) attached to the electrode surface release electrons to become oxygen molecules O_2_, and O_2_ is generated on the electrode [10].

Eq. 5 2H_2_O → 4H^+^ + 4e^−^ + O_2_ ↑

Therefore, bubbles generated on the electrodes during the electrolysis process may be oxygen. In addition, tritiated water is left in the water as tritiated ions (2T) and condensed.

Eq. 6 HTO + H_2_O → 3H^+^ + 2T + 4e^−^ + 2O_2_ ↑

**Fig. 8:**
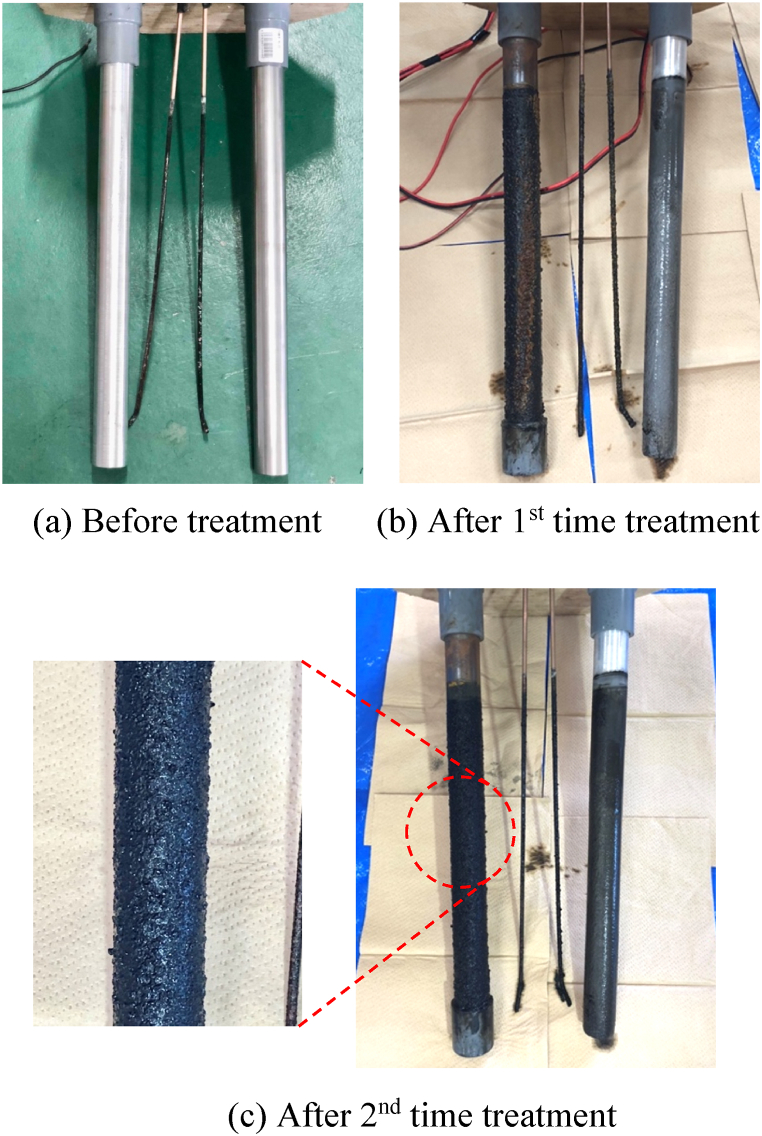
The state of adhered substances on electrodes after the electrolytic treatment. (a) Before treatment (b) After 1st time treatment (c) After 2nd time treatment.

In this treatment, HTO/H_2_O separation is considered by depositing carbide powder (and ore powder), which has adsorbed tritiated water by stirring/circulating treatment as a pretreatment, on electrodes by electrolysis (electrodeposition method), instead of aiming at electrolytic accumulation of tritium by electrolysis of water containing tritium. The state of adhered substances on electrodes after the electrolysis treatment are shown in Fig. 8. Compared to the electrode before treatment (Fig. 8 (a)), an accumulation of additives can be seen on the surface of both electrodes after the 1st treatment (Fig. 8 (b)).

In other preliminary experiments (using 1 L of contaminated water after the same stirring/circulating treatment), the same electrolysis treatment was performed and the tritium radioactivity concentrations of the additives attached to the electrodes and those separated and precipitated were 173 Bq/g and 1498 Bq/g. Therefore, the additives with adsorbed tritium were deposited on the electrodes (or separated and precipitated at the bottom of the electrolytic bath) by the electrolysis treatment, and HTO/H_2_O separation was considered to have been made.

The radioactivity concentration of the tritiated water from the 2nd stirring/circulating treatment was 339 Bq/mL, indicating a decreasing of about 4.50% (about 7.3% above the reference value).

Continuing on, the radioactivity concentration of the tritiated water after the 2nd electrolysis treatment was 333 Bq/mL, further decreased by about 1.17%, and finally showed high HTO/H_2_O separation efficiency of approximately 9.02% compared to the reference value (366 Bq/mL) before treatment. Fig. 8 (c) shows the state of electrodes after the 2nd electrolysis treatment. Adhesion of additives to the electrode was also observed in the 2nd electrolysis treatment.

Furthermore, highly concentrated tritium pseudo-contaminated water was treated in the same process (Fig. 1). The radioactivity concentration of the prepared tritiated pseudo-contaminated water (50 L) before treatment was 1135 Bq/mL. The tritium concentration after treatment was 1060 Bq/mL, indicating a decreasing of about 6.6%, and a relatively high separation efficiency was obtained.

Therefore, the effect of the carbide powder (and ore powder) added in this experiment and the electrolytic treatment showed the usefulness of high separation efficiency as a technology for the separation of tritiated water from water (HTO/H_2_O separation).

Furthermore, we performed to confirm reproducibility experiment by this method for the remove tritium water. The treatment process for reproducibility experiment was performed stirring/circulation treatment for 1 h and electrolytic treatment for 5 h and subsequent leaved it for 4 h. For tritiated water, the treated water was sampled before and after stirring/circulation treatment, and the tritium concentration was measured in each sample to calculate the separation efficiency. The experiment was performed four times, and newly prepared tritium pseudo-contaminated water was used for each experiment. Table 1 shows the change in radioactivity concentration before and after treatment of the tritium-simulated water in each experiment. Separation efficiencies exceeding 5% were obtained in all experiments, and reproducibility was observed. Therefore, it was found that this treatment method achieves high separation efficiency of tritium-contaminated water and has excellent reproducibility.

**Table 1:** The radioactive concentration of tritium-contaminated water after the various treatments.

Number of experiments	Radioactive concentration of tritium-contaminated water (Bq/mL)		Removal rate of tritium $\frac{①-②}{①}$ × 100 (%)
	Before treatment ①	After treatment ②	
1st time	1231 ± 2	1159 ± 2	5.85
2nd time	1301 ± 2	1231 ± 2	5.38
3rd time	1271 ± 2	1202 ± 2	5.43
4th time	1274 ± 2	1203 ± 2	5.57
Average value	1269	1198	5.59

**Fig. 9:**
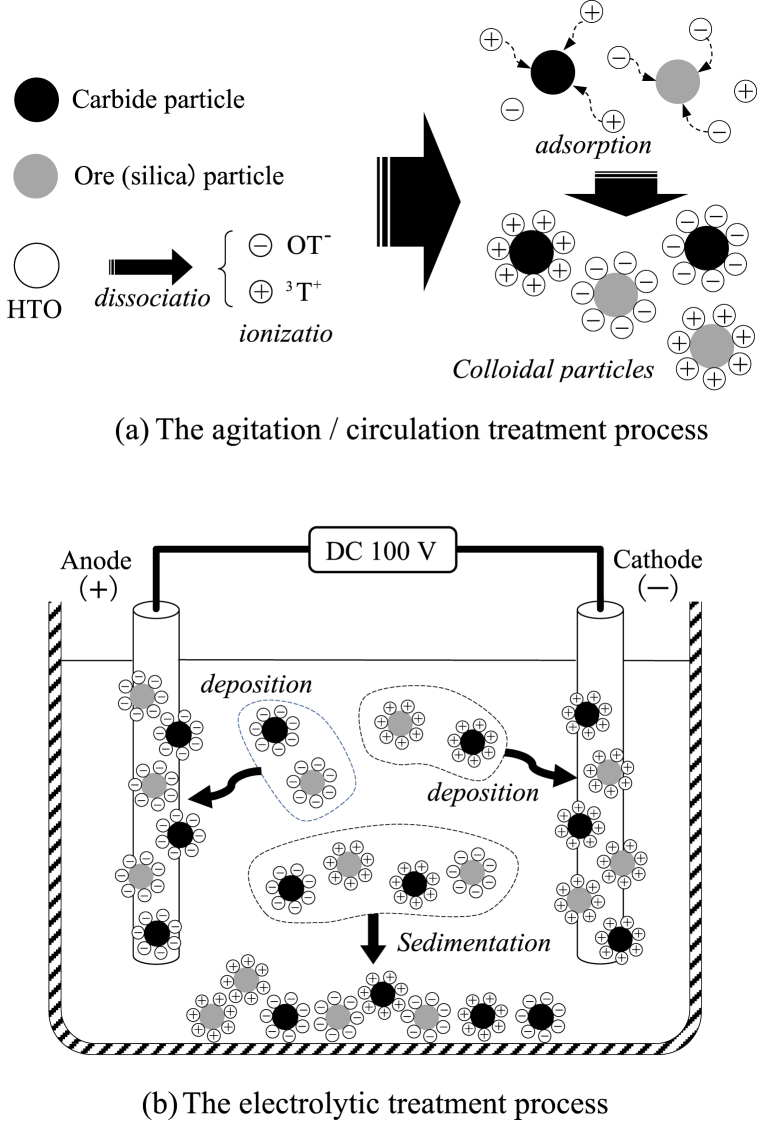
The reaction process of tritium-contaminated water in the various treatment processes. (a) The agitation/circulation treatment process (b) The electrolytic treatment process.

The reaction process of separation/removal of tritiated water (HTO) in each treatment process and the role of additives is shown in Fig. 9. In the stirring/circulating treatment process (Fig. 9 (a)), OT^−^ and ^3^T^+^ are produced by the dissociation and ionization of HTO. The chemical reaction formula of dissociation and ionization of HTO refers to Eqs.3 and 4. These ions are absorbed by particles of carbide or silica ore and generated colloidal particles. Treated HTO containing colloidal particles is transferred to an electrolytic bath for the electrolytic treatment process.

In the electrolytic treatment process (Fig. 9 (b)), colloidal particles in HTO are deposited on the electrodes. Therefore, OT^−^ and ^3^T^+^ were deposited on the anode (+) and the cathode (−), respectively. Part of the colloidal particle sedimented the bottom of the electrolytic bath during the electrolytic treatment. At this time, particles of carbide or silica ore in HTO are also sedimented and HTO after treatment showed a clear and colorless solution as shown in Fig. 6. In addition, part of HTO is generated OT^−^ and ^3^T^+^ ions by the oxygen reduction reaction with the electrodes by the electrolytic treatment process, and a part of their ions are absorbed into particles of carbide or silica ore.

As an advantage of this treatment process; (1) The stirring/circulating treatment process is generated colloidal particles by OT^−^ and ^3^T^+^ by the dissociation and ionization of HTO. (2) The electrolytic treatment process is deposited colloidal particles on the electrodes and can be removed tritium. (3) The additives (powders of carbide and silica ore) are initial nuclei (adsorption substance) to prepare colloidal particles.

These treatment processes using additives were found useful for removal of tritium.

## 4 Conclusion

As a method for separating tritiated water from radioactively contaminated water, we investigated the improvement of separation efficiency by mixing additives into the contaminated water, stirring/circulating the water, and performing electrolysis treatment. The radioactivity concentration of the tritium-contaminated water before treatment was 366 Bq/mL, however after treatment, it was 333 Bq/mL, indicating a decreasing rate of about 9.02% and high separation efficiency was obtained. Separation efficiency of about 6.6% was obtained even for highly concentrated tritium pseudo-contaminated water. In addition, reproducibility experiments yielded separation efficiencies greater than 5% in 4 times experiments. Carbide and ore (silica stone) powders were used as additives and are considered useful as tritium adsorbents. The additives used in this experiment and the treatment method are expected to be used as HTO/H_2_O separation technology.

## Declaration of competing interest

The authors declare that they have no known competing financial interests or personal relationships that could have appeared to influence the work reported in this paper.
